# Paradoxical reactions in ocular tuberculosis

**DOI:** 10.1186/s12348-019-0183-x

**Published:** 2019-09-06

**Authors:** Sudha K. Ganesh, Sharanya Abraham, Sridharan Sudharshan

**Affiliations:** Medical Research Foundation, Sankara Nethralaya, 18, College Road, Chennai, 600 006 India

**Keywords:** Ocular tuberculosis, Paradoxical reaction, Paradoxical worsening, Corticosteroids, Immunosuppressives, Syphilis, HIV, Jarisch–Herxheimer reaction, Immune-reconstitution inflammatory syndrome (IRIS), Anti-tubercular therapy

## Abstract

Paradoxical reactions following initiation of anti-tubercular therapy have been documented most often in extrapulmonary tuberculosis. A combination of factors such as delayed hypersensitivity, decreased suppressor mechanisms, and an increased response to mycobacterial antigens mediated by the host’s immune system have been implicated in the development of these reactions. Similar worsening in patients with ocular tuberculosis while on treatment has been described. It is therefore important for the clinician to be aware of this occurrence, as prompt recognition and timely institution of corticosteroids and immunosuppressants can lead to restoration of vision. In these patients, an alteration or discontinuation of anti-tubercular therapy may not be indicated.

## Introduction

Tuberculosis (TB) is a multi-system disease with protean manifestations. Once infected with the causative organism, *Mycobacterium tuberculosis* (MTB), the individual is at highest risk of developing TB within the first 2 years, but can remain at risk for their lifetime [1]. Nearly one third of the world’s population is infected, with an annual incidence of 126 per 100,000 persons [2]. In the year 2014, 9.6 million people were thought to be infected with TB globally and 1.1 million HIV-negative people died from TB [3]. TB has been declared a global emergency by the World Health Organization (WHO), as it remains the most common cause of mortality from any single infectious disease.

*Ocular TB* may occur due to a direct invasion by TB mycobacteria, as evidenced by the positive culture or histopathology from involved ocular tissue. Most times, however, there may not be clinical or histopathological evidence to suggest active ocular TB infection. The pathogenesis of uveitis in these patients remains unclear and it is not certain, whether the uveitis results from a hypersensitivity response to TB organisms or from reactivation of latent ocular infection by MTB.

With the increase in the global burden of TB, more patients are likely to be diagnosed with ocular TB. Diagnosis of this entity is often presumptive and its management lacks uniform guidelines. Establishing a prior exposure to MTB utilizing tuberculin skin tests (TST), interferon gamma release assays (IGRA), chest X-ray, or high-resolution computed tomography (HRCT) are relatively straightforward. Other techniques used in the detection of active TB, such as culture and lesion biopsy, have limited utility in ocular TB. Multiplex, multi-targeted polymerase chain reaction (PCR) for one or more MTB DNA-coding regions is the most common technique for diagnosis. The diagnosis of ocular TB warrants a strong index of suspicion and, when confirmed with ancillary tests, requires combined anti-inflammatory and anti-tubercular therapy (ATT). Studies from India show that, in patients with vision-threatening uveitis with no identifiable cause and who have latent TB, the recurrence rate of uveitis is markedly reduced with concomitant ATT and uveitis treatment [4]. Similar findings have been demonstrated by studies in Singapore [5], the Netherlands [6], and the UK [7, 8].

### Paradoxical reaction/worsening in TB

This is a well-known entity and its proposed mechanisms include the release of mycobacterial antigens following ATT and delayed hypersensitivity, although the exact pathogenesis is unknown. It is believed to be mediated by the host’s immune system due to a combination of factors including enhanced delayed hypersensitivity, decreased suppressor mechanisms, and increased exposure to mycobacterial antigens or a response to them.

*Definition*: A “paradoxical reaction” while on ATT may consist of clinical or radiological worsening of pre-existing TB lesions or the development of new lesions in patients who showed an initial response or improvement with treatment. Recognition of deterioration that results from a paradoxical reaction rather than that from treatment failure, drug resistance, poor compliance, or secondary diagnoses can be difficult.

### Pathogenesis of paradoxical reactions/worsening in TB infections

Patients identified with a paradoxical worsening generally have a negative TST and decreased lymphocyte blastogenesis at the time of diagnosis, but a positive TST and increased lymphocyte blastogenesis after the initiation of therapy [9–11]. These findings might support the hypothesis of reconstitution of the immune response. Another hypothesis is that the elevation of the tumor necrosis factor-α (TNF-α) level, stimulated by lipoarabinomannan and other lipopolysaccharides in the MTB cell wall, is an initial step in the pathogenesis of paradoxical reactions. The cytokine TNF-α is secreted by macrophages and monocytes, and its increased production and pro-inflammatory activity may play a role in the development of paradoxical worsening [9, 12, 13].

In ocular TB, it is theorized that bacteria-laden macrophages from alveoli may enter the lymphatics and circulation thus carrying the bacteria to the eye, where the organisms may persist and initiate an immune-mediated response [14, 15].

In HIV co-infected patients, paradoxical reactions occur more frequently, with significant reductions in viral load and increase in CD4+ lymphocyte counts after highly active antiretroviral therapy (HAART), and have been reported in 5–35% of patients receiving treatment for TB [16, 17]. In HIV patients with TB co-infection, immune reconstitution inflammatory syndrome (IRIS) is thought to result from a rapid recovery of immune responses against opportunistic pathogens, resulting in a massive inflammatory reaction directed against pathogen-laden tissues. The factors commonly associated with IRIS are a high pathogen burden and an advanced state of immunosuppression, which may be indicated by a very low CD4^+^ T cell count at ART initiation. The rapid expansion of CD4^+^ T-cells specific for the causal opportunistic pathogen, while CD4^+^ T-cells specific for HIV remain stable is an immunological hallmark of IRIS. [18].

Although most cases of paradoxical worsening have been described in adults and children, the paradoxical reaction has been described in a neonate with an immature immune system with congenital TB on ATT. T cell responses in neonates are defective. Cytokine-secreting T cells increase significantly during the first 6 months of life-promoting humoral and cellular immune responses. Therefore, the relative immaturity of immune responses in neonates can be likened to adult patients recovering from deficiencies in cell-mediated immune responses [19].

### Jarisch–Herxheimer reaction (JHR) in systemic disease

Although first described in patients with syphilis, this reaction has also been observed in other bacterial infections. It describes paradoxical worsening following chemotherapy. The reaction is associated with an increase in circulating levels of TNF-α, interleukin-6 (IL-6), and IL-8 and has been reported more frequently among HIV-infected early syphilis patients compared to non-HIV infected controls. Complications due to the reaction have also been described in neurosyphilis, ocular syphilis, and cardiovascular syphilis amounting to the conclusion that systemic corticosteroids might suppress an eventual JHR [20]. The proposed mechanisms of JHR include endotoxin release from the death of organisms, delayed hypersensitivity, and decreased suppressor mechanisms [21]. Apart from syphilis, JHR is common with management of leptospiral infections and Lyme disease. Systemic features of JHR include fever, headache, and sweating. However, in systemic TB, JHR has been described as worsening of intracranial tuberculoma, meningeal disease, tuberculous meningeal radiculitis, pleural effusion, and abdominal TB.

### Paradoxical Reactions/Worsening In Ocular TB

Intraocular TB has varied presentations and may manifest clinically as posterior uveitis, choroidal tubercles or a tuberculoma, subretinal abscess, or serpiginous-like choroiditis; it can also present as retinal vasculitis, granulomatous anterior uveitis, panuveitis, and intermediate uveitis. Paradoxical reaction to ATT and its treatment with escalating doses of steroid and immunosuppressants have been described in various forms of ocular TB. Occurrence of new lesions at the same site or at different sites or the worsening of existing lesions has been described [22, 23].

**Fig. 1:**
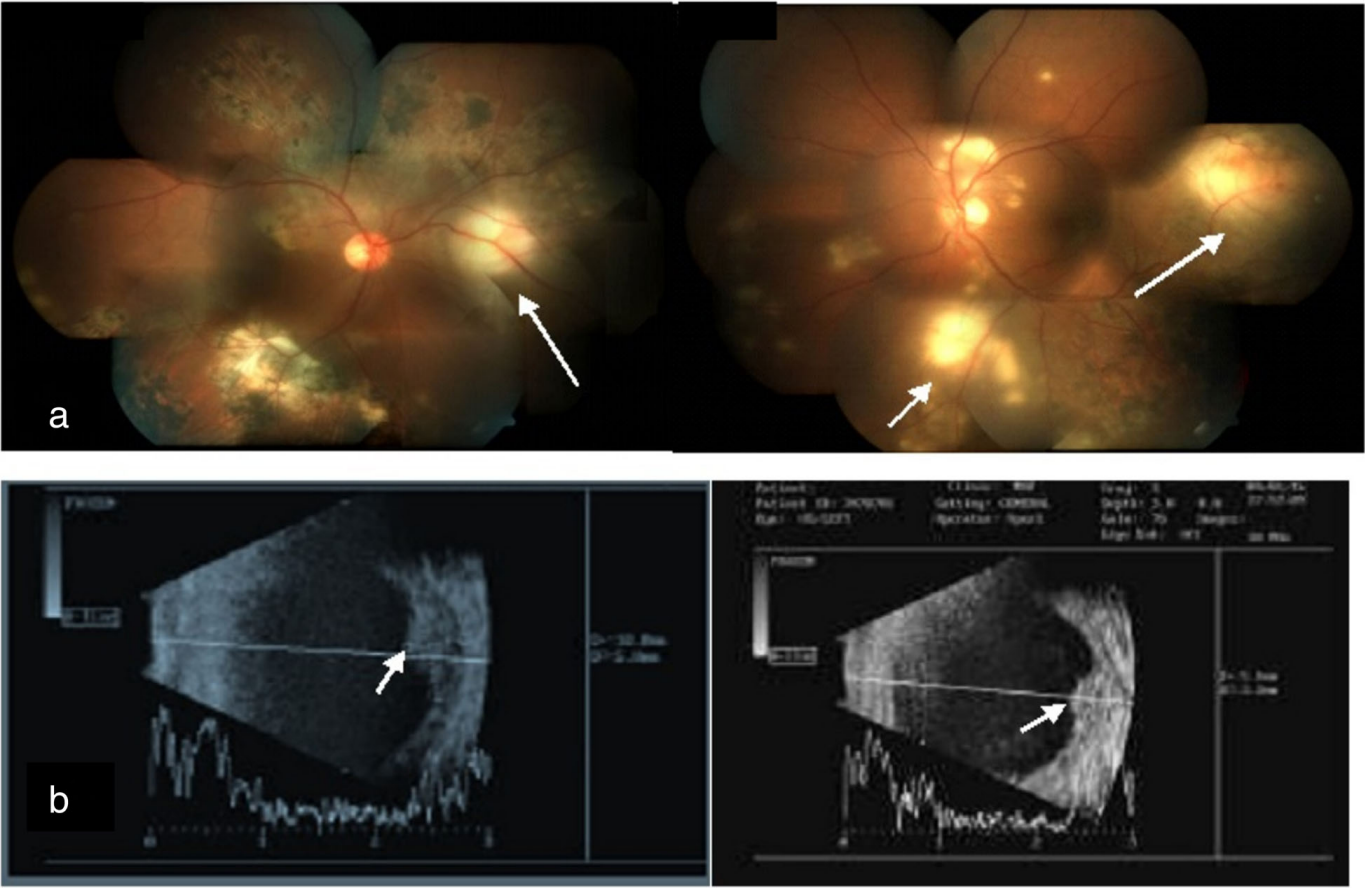
**a** Fundus photograph of both eyes at presentation showing multiple subretinal abscesses with serpiginous-like choroiditis. **b** Corresponding B-scan ultrasonography showing retino-choroidal elevations suggestive of multiple sub-retinal abscesses in both eyes

**Fig. 2:**
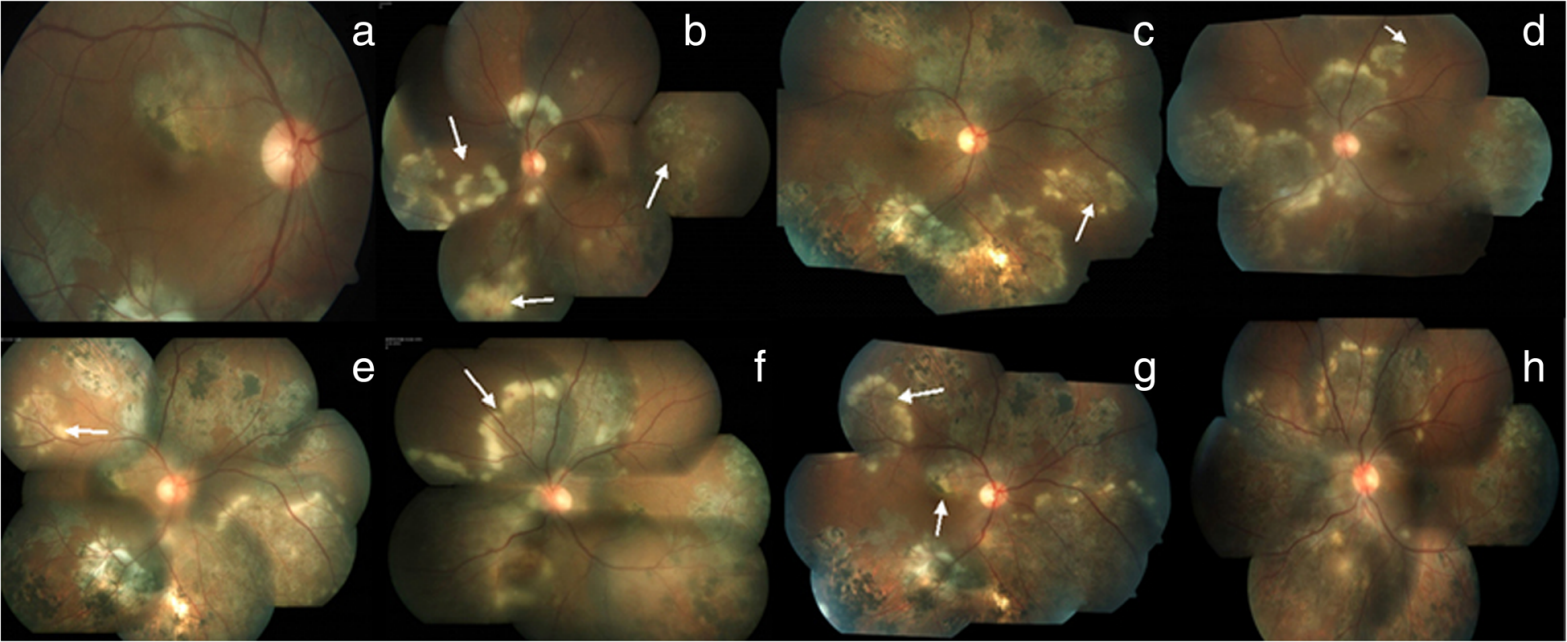
**a**, **b** Fundus photograph at 3 weeks—resolving subretinal abscesses in both eyes, reactivation of serpiginous-like choroiditis in the left eye, a paradoxical reaction. **c**, **d** Fundus photograph at 4 weeks, showing formation of new lesions and progression of old lesions. **e**, **f** Fundus photograph at 6 weeks, showing relentless progression of active lesions and appearance of new lesions. **g**, **h** Fundus photograph at 10 weeks with new active lesions in the right eye involving the fovea

**Fig. 3:**
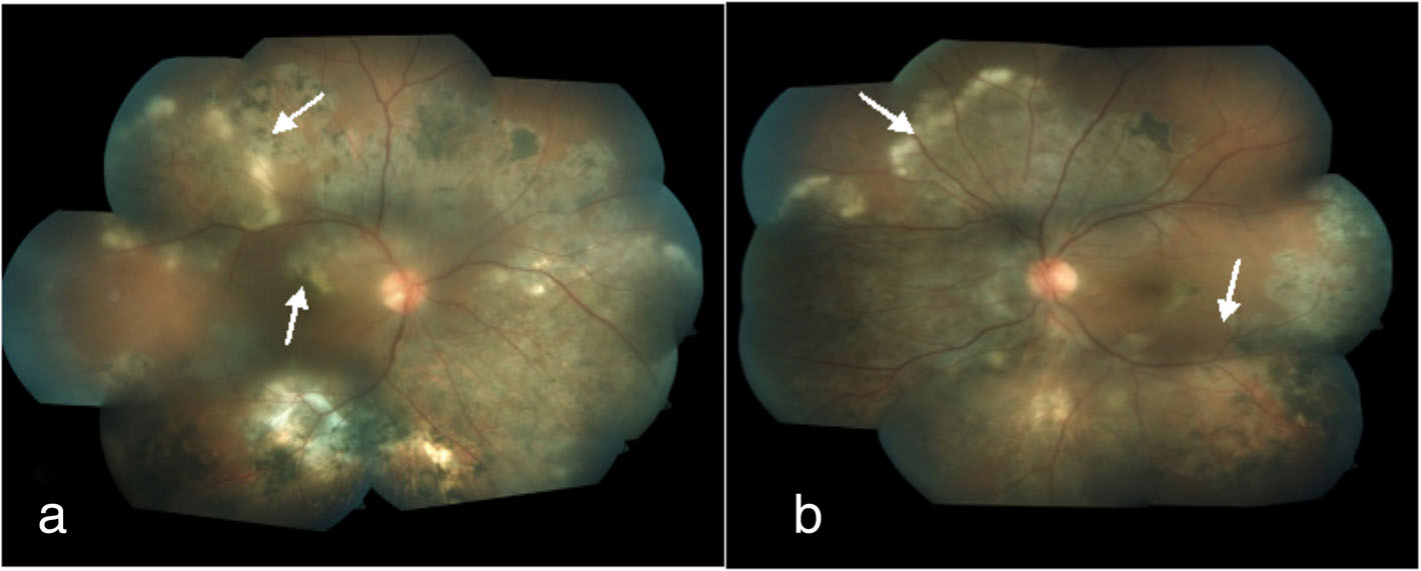
**a**, **b** Fundus photograph of both eyes at 12 weeks showing resolution of the lesions with scarring over fovea in the right eye

In a report by Yilmaz et al., paradoxical worsening of a choroidal tuberculoma was characterized by an enlargement in size seen in a patient with miliary TB [24]. A case report by Cheung and Chee described a patient with biopsy-proven tuberculous cervical lymphadenitis and no ocular findings at the time of diagnosis but developed TB chorioretinitis with paradoxical worsening on ATT [21]. In both reports, treatment with systemic steroid and continuation of ATT yielded good results. A case series by Basu et al. highlighted the pitfalls in the management of TB-associated uveitis, where in two cases, the diagnosis of TB was overlooked at presentation and only steroid was initiated for ocular inflammation. These patients developed intracranial tuberculomas later on along with reactivation of ocular inflammation which then responded well to ATT and systemic steroid. In the subset of patients with serpiginous-like choroiditis, although lesions progressed with ATT, they showed resolution with intravenous methylprednisolone [25]. A similar phenomenon of paradoxical worsening in serpiginous-like choroiditis with similar outcomes has been reported from Turkey by Esen at al. [26]. We have reported a case of bilateral serpiginous-like choroiditis with multiple subretinal abscesses which developed paradoxical worsening [23]. This patient had positive TST and IGRA tests and HRCT chest showed enlarged and calcified mediastinal lymph nodes. Aqueous sampling for PCR for TB was positive. The patient was initially treated with ATT along with oral steroids and showed clinical improvement (Fig. 1). However, this was followed by a paradoxical worsening with new subretinal lesions (Fig. 2). A diagnostic vitrectomy was done and vitreous sample for PCR was also positive for IS6110 genome. A high dose of intravenous corticosteroids and immune-suppressive agents were initiated with a favorable response. The patient responded well to treatment with ATT along with oral corticosteroids and immunosuppressants (Fig. 3). These cases illustrate the possibility of developing a paradoxical reaction while on ATT in different ethnic populations.

To understand the spectrum of phenotypes of choroidal involvement in TB uveitis and geographical variations in disease expression, a retrospective cohort study of patients was conducted in 25 multinational centers. It was found that, out of the 245 patients included, 159 (64.9%) were of Asian origin, 53 (21.6%) were of European/White descent, and 10 (4.1%) of African origin. Regional variation was noted in the phenotypes of choroidal involvement. Serpiginous-like choroiditis (SLC) was found to be the most common presentation in the East (Asia, Australia and the Middle-East), while in the West (Europe, America and Africa), presentation as SLC was second to choroiditis, that did not fit the diagnosis of multifocal choroiditis, APMPPE, choroidal tuberculoma or ampiginous choroiditis. A regional variation in the use of ATT was also noted. This may highlight the occurrence of paradoxical worsening and treatment failures [27].

A paradoxical reaction to ATT while managing ocular TB should be identified early and managed appropriately. A recent prospective, longitudinal study has analyzed the serum cytokine profile in patients with tubercular multifocal serpiginoid choroiditis (TB MSC) receiving ATT and oral steroids. Cytokine analysis was done in two groups: those with and without paradoxical worsening. The study concluded that patients with paradoxical worsening in TB-related uveitis may show a heightened immune response with higher baseline IL-10 values, early rising levels of interferon- ϒ (IFN-ϒ), progressive increase in transforming growth factor-β (TGF-β), and rising levels of TNF-α after initiation of ATT and corticosteroids. Patients with high IL-10 values have low bacilli clearance rates and more chance of persistence of infection [28]. Further, MTB is capable of stimulating the release of IL-10. Thus, higher mean levels of IL-10 at baseline may indicate higher tubercular antigenic load in the body. Therefore, patients who show a progressive rise of TNF-α and rapid rise of IFN-ϒ as early as 1 week after initiation of ATT and corticosteroids and high baseline levels of IL-10 may be at a higher risk of paradoxical worsening and sight-threatening manifestations.

Employing newer imaging modalities has been found to be helpful in identifying paradoxical reaction to ATT while managing ocular TB. Ultra-widefield imaging may be considered more useful than conventional imaging in identifying additional choroiditis lesions and paradoxical worsening, particularly in the retinal periphery that alters the course of therapy in tubercular multifocal serpiginoid choroiditis. A prospective, observational study has shown the presence of central and peripheral paradoxical worsening in 36.4% of patients, adding significant value to diagnosis and management [29].

Retinochoroidal microvasculature has been studied using optical coherence tomography angiography (OCTA) in subjects with TB MSC, who developed paradoxical worsening on the initiation of ATT. The lesions were associated with increased areas of choriocapillaris flow void on en face OCTA in all eyes. There was partial healing in the center and continuous progression at the active edges. Progression of lesions was evidenced by the development of vascular tufts. Thus, with OCTA, it may be possible to grade pathologic changes in the inner choroidal vasculature that take place during the progression of the disease and to identify paradoxical reaction to ATT [30].

### Paradoxical worsening of TB In HIV co-infection

Paradoxical worsening in patients with HIV/AIDS can be due to increased pathogenicity of the organisms due to low immunity, increased viral load, and drug resistance or a result of immune reconstitution inflammatory syndrome (IRIS).

Paradoxical worsening is thought to represent an improvement of the host’s immune response to mycobacterial antigens during the course of treatment, leading to a more intense inflammation at sites of tubercular disease. The incidence of paradoxical worsening of TB in HIV-infected persons was found to be 11% in patients receiving HAART and 7% in patients not receiving antiretroviral therapy (*p* = 0.67). Cases complicated by paradoxical worsening were more likely to have both pulmonary and extrapulmonary disease at initial diagnosis than cases without paradoxical worsening (83% versus 24%; *p* = 0.006). TB relapse occurred in 33% of patients with paradoxical worsening and in 5% of patients without paradoxical worsening (*p* = 0.06), signifying the need to identify risk factors and appropriate duration of ATT in these patients [31].

Although an essential, life-saving intervention for HIV infection, anti-retroviral therapy (ART) can frequently be complicated by tuberculosis-associated immune reconstitution inflammatory syndrome (TB-IRIS) in a TB endemic setting. The incidence of paradoxical TB-IRIS is estimated at 18% (95% CI 16–21%), higher than previously reported and over 50% in high-risk groups. Although early ART initiation in TB patients with CD4+ counts less than 50 cells/mm^3^ improves survival, it increases TB-IRIS risk by greater than twofold [32]. As recommended in Table 1, patients with lower counts should be initiated on ATT at the earliest [34].

**Table 1:** Commencement of ART in TB infected individuals [33]

CD4 count (cells/mm^3^)	Initiation of ART
< 50	Early ART within 2 weeks of ATT
> 50	Between 4 and 8 weeks of ATT
≥ 200	ART recommended but commencement is less urgent

**Fig. 4:**
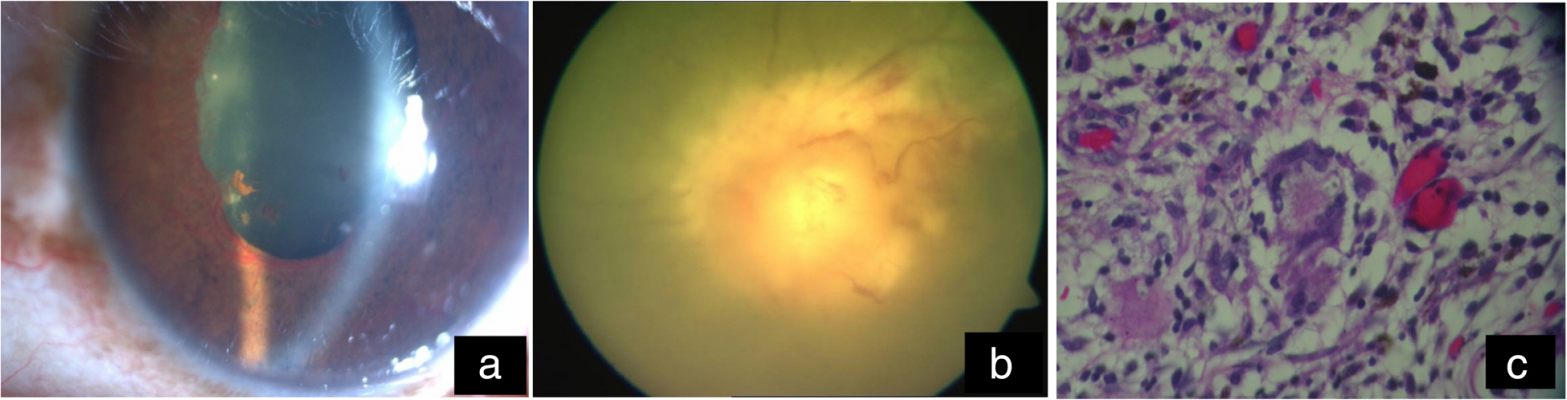
**a**, **b** Progression to panophthalmitis despite improving CD4 counts. **c** Histopathology of eviscerated tissue showing typical caseation necrosis with epithelioid cells and Langhans giant cells

Studies on TB-IRIS imply high antigen burden, innate immune cell cytotoxicity, inflammasome activation, and dysregulated matrix metalloproteinases in the pathogenesis of the condition. Clinical worsening following ATT in patients with HIV and ocular TB has been commonly reported to present as panophthalmitis. We have reported a series of ocular TB in HIV/AIDS from our center, in which 3 out of 7 cases that presented as subretinal abscess progressed to panophthalmitis [35]. Poor immune status as evidenced by low CD4 counts may have been responsible for such exuberant reactions. However, in this series, panophthalmitis occurred in one patient with a higher CD4 cell count. This patient was started on HAART prior to ATT. The elevation of CD4 cell count from 263 to 560 cells/μl led to immune reconstitution phenomena followed by worsening of intraocular inflammation. This case represents the emerging phenomenon of paradoxical worsening of tubercular infection following the initiation of HAART (Fig. 4a, b).

Both histopathologic and microbiological examination of the eviscerated tissue in our reported series revealed plenty of acid-fast bacilli indicating a severe intraocular infection with MTB (Fig. 4c). Panophthalmitis may have occurred due to impaired cell-mediated immunity in the patient as a result of HIV infection. HIV-TB co-infection enhances the pathogenicity of MTB, causing florid inflammation leading to panophthalmitis and abscess formation.

Worsening of subretinal abscesses in IRIS or paradoxical worsening are most commonly reported in the literature. IRIS also leads to worsening of signs and symptoms in HIV/AIDS patients with systemic TB [36]. Paradoxical worsening leading to globe perforation in a patient receiving HAART along with ATT has been reported [37].

Alternative management strategies such as delaying the administration of HAART for the first 2 months of ATT have been suggested with the aim of increasing adherence to both therapies [38].

Immune recovery uveitis (IRU) is the most common form of IRIS in HIV-infected patients with cytomegalovirus (CMV) retinitis who are receiving HAART. IRU is presumed to be mediated by the recovery of immune responses specific to residual CMV antigen located in the eye. In addition to improved immunity itself, risk factors include a low CD4 T count at the time of initiation of HAART and involvement of a larger proportion of retina [39].

A case of massive mycobacterial choroiditis during HAART, attributable to *Mycobacterium avium* complex (MAC) has also been reported [40]. Pathogenesis of this entity was related to an enhanced immune response to a prior subclinical, disseminated infection. The formation of discrete granulomas that are normally absent in MAC infections in AIDS reflects this mechanism. In patients with AIDS, MAC is the most common cause of systemic bacterial infections in the USA. Rosenbaum and associates have described a patient with end-stage AIDS and disseminated MAC presenting with iris nodules as the initial manifestation of panophthalmitis [41].

### Management

As there are no recent randomized trials conducted for the optimum treatment for ocular TB, the management followed is similar to that recommended by The American Thoracic Society (ATS), the Centres for Disease Control (CDC), and the Infectious Diseases Society of America (IDSA). They recommend four drugs: isoniazid (INH), pyrazinamide (PZA), ethambutol (EMB), and rifampicin (RIF) for an initial 8 weeks followed by INH and RIF either 7 days a week (regimen 1a) or twice weekly (regimen1b) for a minimum of 18 weeks. These principles are applicable to HIV-infected patients in whom similar regimens of four drugs for a period of 6–9 months are found to be as effective. The World Health Organization (WHO) also recommends the use of four drugs (INH/RIF/PZA/ETB) for an initial 2 months followed by INH/RIF for 4 months for category I patients (new sputum-positive patients, new sputum-negative patients who have extensive lung parenchymal disease, and those with severe extrapulmonary disease) as well as patients in category III (new smear-negative patients with less lung parenchymal involvement and patients with less severe extrapulmonary disease) [42].

In all cases of active intraocular TB, treatment with ATT may be warranted, similar to that for pulmonary TB as per CDC recommendations for active systemic TB. Choroidal tubercles and tuberculomas generally show a good response to ATT without any concomitant treatment. However, when TB-associated choroiditis is treated with ATT, there may be an initial paradoxical worsening; concomitant treatment with oral corticosteroids has been considered to circumvent this phenomenon. Systemic corticosteroids used in conjunction with ATT also help to prevent further damage to ocular tissues from the inflammatory response [14]. However, corticosteroid treatment alone can cause further progression of the infection and so should not be used without concomitant ATT. Consultation with a pulmonologist or infectious diseases specialist is indicated prior to starting ATT for ocular lesions. A good knowledge of available first and second-line ATT, their dose, side effects, drug interactions, and recognition of multidrug resistance is essential prior to the institution [43].

Tubercular anterior uveitis is treated with topical cycloplegics and topical corticosteroids, along with ATT [44]. Oral, depot, or topical corticosteroids may also occasionally be used in addition to ATT, in the treatment of intermediate uveitis in order to prevent cystoid macular edema (CMO). Management of retinal vasculitis involves both ATT and oral corticosteroids, along with laser photocoagulation of the non-perfused retina [14, 44].

The difference in practice pattern of treating TB-related uveitis depends on geographic location, prevalence of TB, and personal experience in treating TB-related uveitis [43].

Alternate regimens may depend on regional resistance patterns, and duration of treatment can be extended in the setting of multi-drug resistance or slow response to treatment [14]. A meta-analysis has shown that 84% (95% CI 79–89) of the patients receiving ATT showed non-recurrence of inflammation during the follow-up period, 69% (95% CI 33–96) had an improvement in visual acuity, and 92% (95% CI 63–100) showed an improvement in inflammation [43]. In a cohort of patients attending uveitis clinics in London and Sydney, it was found that treatment with ATT halved the risk of uveitis recurrence and delayed the onset of the first recurrence in eyes with uveitis associated with latent TB [45].

In general, patients who show a response within 2 months may benefit with 6 months of ATT. In those without a response at 2–3 months, there may be a need to identify a second line of therapy or change treatment based on the overall health of the patient and consultation with an infectious diseases specialist [43]. Other adjuncts to treatment such as rifabutin, fluoroquinolones, interferon gamma, and linezolid may be considered in cases of multidrug resistance [14].

Basu and colleagues [22], in their retrospective case series of 147 patients, found the progression of active inflammation after initiation of ATT in 26 patients (24.5%), and the majority were seen in intermediate uveitis (37%). They also reported worsening in cases of granulomatous anterior uveitis, retinal vasculitis, serpiginous like-choroiditis (25.9%), and panuveitis. It was found that all patients responded well to escalating doses of corticosteroids. Bansal and colleagues [46] in their case series of 110 patients with SLC reported progression of lesions with ATT in 14% of patients managed with ATT, steroids and immunosuppressives in 3 patients. The similarity of RPE cells to macrophages causing cytokine release with antibiotic therapy was their proposed reason. Siantar and colleagues [47] have reported a case of paradoxical reaction in a patient with tuberculous chorioretinitis on ATT that was treated with oral steroids. Although there is no consensus, the timing of initiation of steroid therapy while on ATT has been addressed taking into consideration the fact that rifampicin is known to decrease the bioavailability of prednisolone by 66% [47, 48].

There is limited experience regarding the use of local immunosuppression in TB uveitis.

Fonollosa et al. have described the use of local dexamethasone implant for the management of paradoxical worsening of MSC lesions [49]. Intravitreal dexamethasone implant for active uveitis with CMO, MSC, retinal vasculitis, and in those with intolerance to systemic steroid has been described. The safety and efficacy of this agent as an adjunct with ATT was also analyzed, showing it was well tolerated without any adverse side effects [50].

The use of a single injection of intravitreal methotrexate in macula threatening MSC as an adjunct with systemic ATT has been described [51].

Intravitreal bevacizumab in two doses 1 month apart showed marked improvement in both the functional and anatomical outcomes in a HIV patient with choroidal granuloma. In this patient, the reversal of the vascular permeability by intravitreal bevacizumab played a role in the reversal of the serous retinal detachment. Phase II trials of bevacizumab in well-controlled HIV patients have shown that it can be safely administered; however, data on immune-deficient individuals is awaited [52].

Drug resistance should be taken into account while managing patients with paradoxical worsening. The GeneXpert MTB/RIF assay is a novel integrated diagnostic device that performs sample processing and heminested real-time PCR (RT-PCR) analysis of the 81-bp fragment of the rpoB gene for diagnosis of TB and rapid detection of rifampicin (RIF) resistance in clinical specimens [53]. Line probe assay (LPA) can be performed directly from acid-fast bacilli (AFB) smear-positive sputum, or from culture isolates, providing results in 1–2 days. Recent studies have concluded that LPAs are highly sensitive and specific for detection of RIF resistance (≥ 97% and ≥ 99%) and isoniazid resistance (≥ 90% and ≥ 99%) on culture isolates and smear-positive sputum [54].

Systemic anti-inflammatory therapy especially corticosteroids initiated alongside ART in selected patients with CD4 less than 100 cells/mm^3^ reduced the risk of paradoxical TB-IRIS by 30% and was not associated with significant adverse effects. Further, corticosteroids remain the mainstay therapeutic intervention for TB-IRIS.

All patients on ATT should be on close follow-up for signs of paradoxical worsening of TB. This may be evidenced as clinical or radiological worsening or the development of new lesions at the same or different site. If any of these changes are observed, the possibility of a recurrence due to insufficient anti-inflammatory therapy/paradoxical worsening, or an incorrect diagnosis of TB/other causes of infectious uveitis, or development of drug resistance to ATT should be considered.

**Fig. 5:**
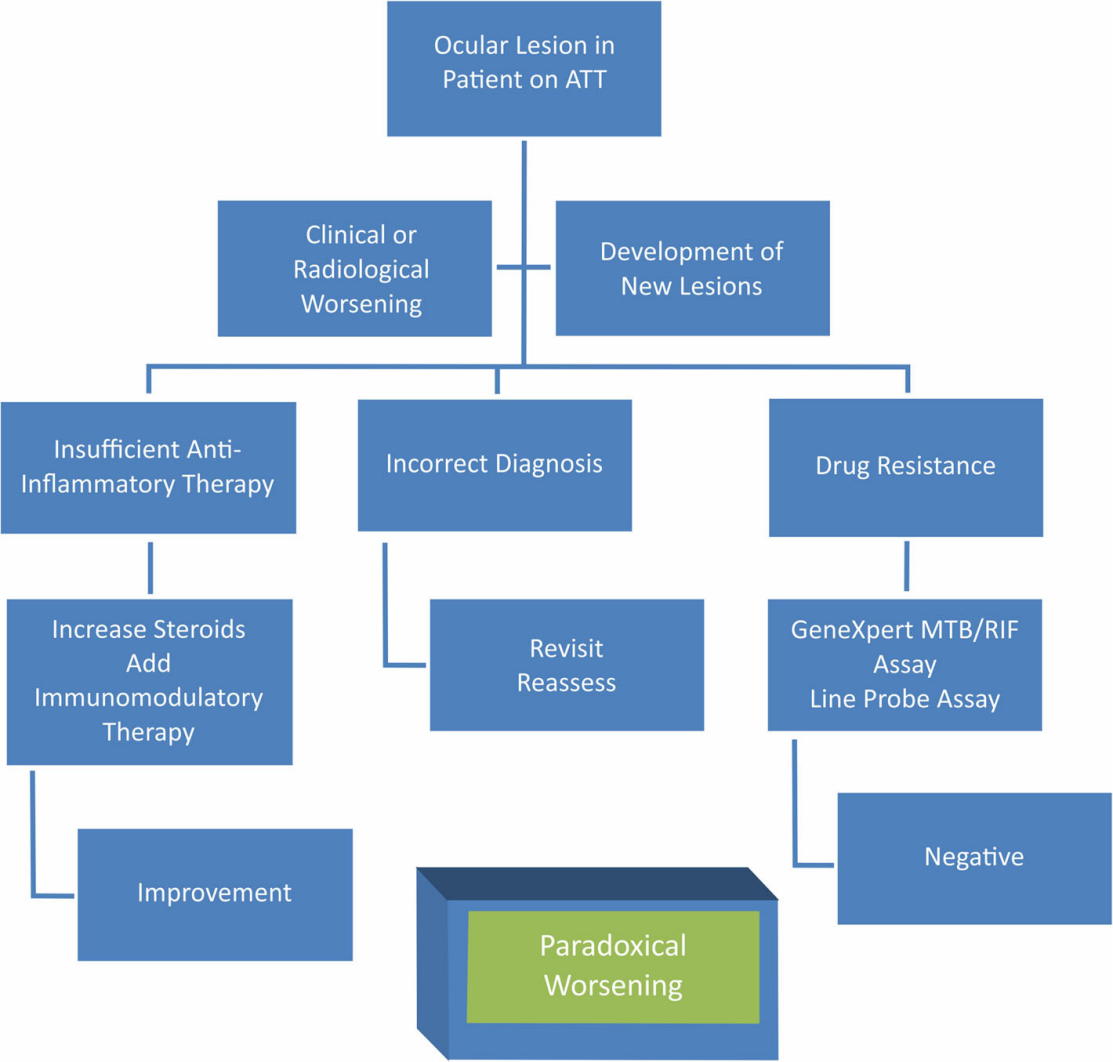
Paradoxical worsening of TB—a management algorithm

A step-wise approach to a patient with ocular TB on ATT has been illustrated (Fig. 5).

- If the worsening is due to insufficient therapy/paradoxical worsening, an increase in the dose of steroid or change in its route of administration along with the addition of immunosuppressives should work to reduce the inflammation.
- If the patient does not respond adequately, other causes of infectious uveitis and testing for the same by PCR/RT-PCR techniques must be undertaken and the patient should be managed appropriately.
- In the setting of proven TB and an initial adequate response followed by worsening or non-response to ATT and anti-inflammatory therapy, a differential diagnosis of drug-resistance must be considered. In these patients, additional tests for drug resistance such as GeneXpert MTB/RIF assay and line probe assay (LPA) may be performed and the ATT regimen altered accordingly.

Thus, in patients with a true paradoxical worsening, as shown in literature, a clinical improvement will be seen with the increase in the dose of steroid or change in route of administration and/or addition of immunosuppressives. However, ATT may be continued uninterrupted and according to schedule.

## Conclusion

Paradoxical reactions and IRIS constitute a spectrum of clinical presentations that occur with the management of MTB infections. The understanding of the fundamental processes shared between the syndromes and their underlying molecular mechanisms may help in the development of appropriate immunotherapy for PR/IRIS and may also delineate the role of inflammation and immunodeficiency in MTB infections. It is therefore important for the clinician to be aware of this occurrence, as prompt recognition and timely institution of corticosteroids and immunosuppressants can lead to the restoration of vision. In these patients, an alteration or discontinuation of anti-tubercular therapy may not be indicated.

## Methodology

In this review, we have evaluated the available literature on paradoxical reactions/worsening in TB, and we have attempted an overview of the clinical features and management of paradoxical reactions in ocular TB in immunocompetent and immunosuppressed individuals. A search of articles using the MEDLINE database and PubMed (National Library of Medicine) was performed to identify all relevant articles published in the field. Terms and phrases used for the search included ocular tuberculosis, paradoxical reaction, paradoxical worsening, corticosteroids, immunosuppressives, syphilis, HIV, Jarisch–Herxheimer reaction, immune-reconstitution inflammatory syndrome (IRIS), and anti-tubercular therapy. Articles were included if they were in English and if access to them could be obtained. Articles included systematic reviews, randomized controlled trials (RCTs), and cohort study data. Review articles, case reports, and editorials were also included if evidence was suitable for inclusion.

## Data Availability

Data sharing is not applicable to this review article as no datasets were generated or analyzed during the current study

## Abbreviations

AFB: Acid fast bacilli
ART: Anti-retroviral therapy
ATS: American Thoracic Society
ATT: Anti-tubercular therapy
CDC: Centers for Disease Control
CMO: Cystoid macular edema
CMV: Cytomegalovirus
CT: Computed tomography
EMB: Ethambutol
HAART: Highly active antiretroviral therapy
HIV: Human immune deficiency virus
HRCT: High-resolution CT
IDSA: Infectious Diseases Society of America
IFN-ϒ: Interferon gamma
IGRA: Interferon gamma release assays
IL-10: Interleukin-10
INH: Isoniazid
IRIS: Immune reconstitution inflammatory syndrome
IRU: Immune recovery uveitis
JHR: Jarisch–Herxheimer reaction
LPA: Line probe assay
LTBI: Latent tuberculosis infection
MAC: *Mycobacterium avium* complex
MDR: Multidrug resistant
MTB: *Mycobacterium tuberculosis*
PCR: Polymerase chain reaction
PZA: Pyrazinamide
RIF: Rifampicin
RPE: Retinal pigment epithelial
RT-PCR: Real-time PCR
SLC: Serpiginous-like choroiditis
TB MSC: Tubercular multifocal serpiginoid choroiditis
TB: Tuberculosis
TB-IRIS: Tuberculosis-associated immune reconstitution inflammatory syndrome
TGF-β: Transforming growth factor-β
TNF-α: Tumor necrosis factor-α
TST: Tuberculin skin tests
WHO: World Health Organization
